# Siponimod (BAF312) prevents synaptic neurodegeneration in experimental multiple sclerosis

**DOI:** 10.1186/s12974-016-0686-4

**Published:** 2016-08-26

**Authors:** Antonietta Gentile, Alessandra Musella, Silvia Bullitta, Diego Fresegna, Francesca De Vito, Roberta Fantozzi, Eleonora Piras, Francesca Gargano, Giovanna Borsellino, Luca Battistini, Anna Schubart, Georgia Mandolesi, Diego Centonze

**Affiliations:** 1 Laboratory of Neuroimmunology and Synaptic Transmission, IRCCS Fondazione Santa Lucia, Centro Europeo di Ricerca sul Cervello (CERC), 00143 Rome, Italy; 2 Multiple Sclerosis Research Unit, Department of Systems Medicine, Tor Vergata University, 00133 Rome, Italy; 3 Unit of Neurology and Neurorehabilitation, IRCCS Istituto Neurologico Mediterraneo (INM) Neuromed, 86077 Pozzilli, IS Italy; 4 Neuroimmunology Unit, IRCCS Fondazione Santa Lucia-CERC, 00143 Rome, Italy; 5 Novartis Institutes of Biomedical Research, Basel, Switzerland

**Keywords:** Striatum, Synaptic transmission, GABA, Neurodegeneration, Experimental autoimmune encephalomyelitis, Parvalbumin neuron

## Abstract

**Background:**

Data from multiple sclerosis (MS) and the MS rodent model, experimental autoimmune encephalomyelitis (EAE), highlighted an inflammation-dependent synaptopathy at the basis of the neurodegenerative damage causing irreversible disability in these disorders. This synaptopathy is characterized by an imbalance between glutamatergic and GABAergic transmission and has been proposed to be a potential therapeutic target.

Siponimod (BAF312), a selective sphingosine 1-phosphate1,5 receptor modulator, is currently under investigation in a clinical trial in secondary progressive MS patients. We investigated whether siponimod, in addition to its peripheral immune modulation, may exert direct neuroprotective effects in the central nervous system (CNS) of mice with chronic progressive EAE.

**Methods:**

Minipumps allowing continuous intracerebroventricular (icv) infusion of siponimod for 4 weeks were implanted into C57BL/6 mice subjected to MOG_35-55_-induced EAE. Electrophysiology, immunohistochemistry, western blot, qPCR experiments, and peripheral lymphocyte counts were performed. In addition, the effect of siponimod on activated microglia was assessed in vitro to confirm the direct effect of the drug on CNS-resident immune cells.

**Results:**

Siponimod administration (0.45 μg/day) induced a significant beneficial effect on EAE clinical scores with minimal effect on peripheral lymphocyte counts. Siponimod rescued defective GABAergic transmission in the striatum of EAE, without correcting the EAE-induced alterations of glutamatergic transmission. We observed a significant attenuation of astrogliosis and microgliosis together with reduced lymphocyte infiltration in the striatum of EAE mice treated with siponimod. Interestingly, siponimod reduced the release of IL-6 and RANTES from activated microglial cells in vitro, which might explain the reduced lymphocyte infiltration. Furthermore, the loss of parvalbumin-positive (PV+) GABAergic interneurons typical of EAE brains was rescued by siponimod treatment, providing a plausible explanation of the selective effects of this drug on inhibitory synaptic transmission.

**Conclusions:**

Altogether, our results show that siponimod has neuroprotective effects in the CNS of EAE mice, which are likely independent of its peripheral immune effect, suggesting that this drug could be effective in limiting neurodegenerative pathological processes in MS.

## Background

Multiple sclerosis (MS) is an inflammatory neurodegenerative disease of the central nervous system (CNS), affecting young adults. Clinically, two main forms of MS can be distinguished, the relapsing-remitting (RRMS) and the progressive, where the latter is described as the gradual progression of clinical disability in a patient either with a preceding relapsing course (secondary progressive MS (SPMS)) or without a preceding relapsing course (primary progressive MS (PPMS)) [1].

Such phenotypic distinction seems to rely on predominance of distinct pathogenic mechanisms that appear different, though poorly understood, throughout RRMS, PPMS, and SPMS [2]. In general, although inflammation has a recognized pivotal role in triggering the cascade of events leading to both white and gray matter (GM) damage, anti-inflammatory or immunomodulatory drugs have been found successful only in RRMS [3], with the recent exception of ocrelizumab, which was found to reduce disability progression also in PPMS [4]. A forthcoming challenge is therefore to identify drugs able to interfere with the mechanisms leading to neurodegeneration in PPMS and in SPMS.

GM pathology in MS is increasingly recognized to contribute to the progression of the disease. In fact, GM damage occurs early in the disease course, is independent of demyelination, and is associated with neurological and neuropsychological disability [5]. Several studies in experimental autoimmune encephalomyelitis (EAE), the murine model of MS, have underscored an inflammation-dependent synaptopathy affecting different brain structures (striatum, hippocampus, cerebellum) and occurring independently of demyelination [6–10]. In such brain areas of EAE mice, the excitatory glutamatergic transmission is potentiated, while the GABAergic inhibitory transmission is reduced: this imbalance in synaptic transmission leads to excitotoxicity and subsequently to neurodegeneration [11]. Of note, glutamate receptor antagonists and gamma-amino butyric acid (GABA) agonists exert beneficial effects in both EAE and MS [12–16]. This synaptopathy has been proposed as a valuable therapeutic target [11].

Several drugs are under investigation for their neuroprotective effects in progressive MS. In particular, an ongoing phase-III clinical trial is currently conducted with the sphingosine-1-phosphate receptor (S1P) modulator, siponimod (BAF312) [17]. Siponimod is a next-generation S1P modulator selective for S1P1 and S1P5 that showed good safety and tolerability in humans. This compound has recently successfully passed a phase-II clinical trial in RRMS [18].

Compared to fingolimod, an S1P modulator selective for S1P1, S1P3, S1P4, and S1P5, the half-life of siponimod is shorter, allowing recovery of peripheral lymphocyte count to a normal range within 1 week after treatment cessation. The risk of bradycardia is mitigated using a dose titration scheme during treatment initiation [19, 20].

Most of the effects of siponomid are attributed to S1P1 expressed on lymphocytes, thereby preventing lymphocyte trafficking into the brain, but both S1P1 and S1P5 are widely expressed in brain-resident cells, like neurons, microglia, astroglia, and oligodendrocytes [21].

The aim of the present study was therefore to assess the central effects of siponimod in mice with myelin oligodendrocyte glycoprotein peptide 35-55 (MOG_35-55_)-induced EAE, a model of progressive MS, to provide a substrate of the putative neuroprotective effects of this drug.

## Methods

### EAE model

EAE was induced as described previously [7, 20]. Mice were injected subcutaneously at the flanks with 200 μg of MOG_35-55_ emulsion to induce EAE by active immunization. The emulsion was prepared under sterile conditions using MOG_35-55_ (85 % purity; Espikem) in 300 μl of complete Freund’s adjuvant (CFA; Difco) containing *Mycobacterium tuberculosis* (8 mg/ml, strain H37Ra; Difco) and emulsified with phosphate buffer solution (PBS). All animals were injected with 500 ng of pertussis toxin (Sigma) intravenously on the day of immunization and 2 days later. Control animals received the same treatment as EAE mice without the immunogen MOG peptide, including complete CFA and pertussis toxin (referred to as hereafter as “CFA”). Animals were scored daily for clinical symptoms of EAE according to the following scale: 0 = no clinical signs, 1 = flaccid tail, 2 = hindlimb weakness, 3 = hindlimb paresis, 4 = complete bilateral hindlimb paralysis, and 5 = death due to EAE; intermediate clinical signs were scored by adding 0.5 [15, 22]. For each animal, the onset day was recorded as the day post-immunization (dpi) when it showed the first clinical manifestations.

Experiments were carried out in accordance with Internal Institutional Review Committee, the European Directive 2010/63/EU and the European Recommendations 526/2007, and the Italian D.Lgs26/2014. All the efforts were made to minimize the number of animals utilized and their suffering.

### Siponimod formulation for minipump and surgery

Siponimod (Novartis Pharma AG) was dissolved in a solution containing 10 % Solutol/Kolliphor HS15 (BASF Pharma Solutions)—final pH range between 6 and 7—at a final concentration of 2 mg/ml. This preparation allowed stability of the drug for up to 6 weeks at 37 °C.

One week before immunization, mice were implanted with subcutaneous osmotic minipumps allowing continuous intracerebroventricular (icv) infusion of either vehicle or siponimod for 4 weeks (three sets of immunizations) [8, 22]. Different siponimod dosages were tested: 4.5, 0.45, and 0.225 μg/day.

### T cell absolute count

T cell absolute count was performed on blood samples kept from the mandibular vein of the mouse.

For the phenotypic characterization of cell populations, the following antibodies were used: CD8-FITC (Miltenyi Biotec), CD25-APC (Pharmingen), CD3-PE-Vio770 (Miltenyi Biotec), CD4-APC-Vio770 (Milteny Biotec), CD45R (B220)-Violblu (Milteny Biotec), NK1.1-PE (Milteny Biotec).

At predetermined optimal concentrations, 100 μl of blood was stained by incubation with the antibodies. Fifty microliters of CountBright Absolute Counting Beads (Molecular Probes) was added, and, following lysis of red blood cells, cells were acquired on a CyAn Cytometer (Beckman Coulter). By comparing the ratio of bead events to cell events, absolute numbers of cells in the sample were calculated.

Some experiments were performed by acquiring the stained blood samples on the CytoFLEX cytometer (Coulter), equipped with a volumetric sample injection module, which enables volumetric sampling and provides absolute cell counts for all samples without the use of beads.

### Determination of siponimod in mouse blood by LC-MS

For quantitative determination, 10 spiked samples from 0.5 up to 10,000 ng/ml were prepared in the same matrix. Proteins were removed by protein precipitation by adding an organic solvent mixture. The organic layer was evaporated to dryness, and the residue was re-dissolved in HPLC buffer B, containing 5 mM ammonium formate.

Aliquots of 2 μl were directly injected on a Agilent Eclipse Plus, RRHD 2.1 × 50 mm reversed-phase column with 1.8-μm particles, and kept at 40 °C.

For separation, a linear gradient from 50 to 100 % B within 1.7 min was used. Solvent A was 0.2 % formic acid in water and solvent B 0.2 % formic acid in acetonitrile. The flow was kept at 500 μl/min during the whole cycle.

For detection, the column effluent was guided directly to the electrospray source of the Agilent 6490 triple quadrupole MS with parameters optimized for siponimod. Compound and a structure-related internal standard were detected as their [MH]+ ions with the multiple reaction monitoring (MRM) transition 517.3 → 159.0, 416.1 for siponimod. For data processing, the compound to internal standard ratio of the extracted ion chromatograms was used.

The calculation was based on a second order fitted and 1/*x* weighted calibration curve (*r*^2^ = 0.9987). The accuracy of the 10 individually prepared calibration samples was better than 15 %. The recovery of the compounds was 94.3 % (RSD = 1.3 %). The precision of the control samples (*n* = 3), distributed over the whole series, was better than RSD = 1.8 %. The LOQ for siponimod was found to be 0.5 ng/ml, based on the lowest calibration sample.

### Electrophysiology

Mice were killed by cervical dislocation, and corticostriatal coronal slices (200 μm) were prepared from fresh tissue blocks of the brain with the use of a vibratome [6, 15]. Single slices were then transferred to a recording chamber and submerged in a continuously flowing oxygenated artificial cerebrospinal fluid (ACSF) (34 °C, 2–3 ml/min) gassed with 95 % O_2_–5 % CO_2_. The composition of the control ACSF was (in mM) as follows: 126 NaCl, 2.5 KCl, 1.2 MgCl2, 1.2 NaH2PO4, 2.4 CaCl2, 11 Glucose, 25 NaHCO3. Whole-cell patch clamp recordings were made with borosilicate glass pipettes (1.8 mm o.d.; 4–8 MΩ), in voltage-clamp mode, at the holding potential (HP) of −80 mV. Spontaneous excitatory and inhibitory post-synaptic currents (EPSCs, IPSCs) were recorded from medium spiny neurons (MSNs) as in Centonze et al. [15] and Rossi et al. [6]. Siponimod (1 μM) was added to the bath solution for 1 h, before recording. Synaptic events were stored by using P-CLAMP 9.2 (Axon Instruments) and analyzed offline on a personal computer with Mini Analysis 5.1 (Synaptosoft, Leonia, NJ, USA) software. Offline analysis was performed on spontaneous synaptic events recorded during fixed time epochs (1–2 min, three to five samplings), sampled every 5 or 10 min. One to six cells per animal were recorded, and four animals were sacrificed for each experimental group.

### Western blot

Animals were killed by cervical dislocation at 24 dpi. Striata were quickly removed, snap frozen in dry ice, and homogenized as in Mandolesi et al. [8] and Gentile et al. [22]. Soon after blocking with 5 % milk in Tris buffered solution (TBS), the membrane was incubated for 1 h with mouse anti-β-actin primary antibody (1:20,000; Sigma-Aldrich) followed by anti-mouse IgG HRP (1:10,000, GE Healthcare, formerly Amersham Biosciences) secondary antibody. Immunodetection was performed by ECL reagent (Amersham) and membrane was exposed to film (Amersham). Next, a mouse anti-glial fibrillary amino acid protein (GFAP, 1:2000, Immunological Science, over night) primary antibody was used in combination with anti-mouse IgG HRP (1:4000 for GFAP; GE Healthcare, formerly Amersham Biosciences) secondary antibody. Immunodetection was performed as for β-actin. Densitometric analysis of bands was performed by NIH ImageJ software (http://rsb.info.nih.gov/ij/). Western blot (WB) results are presented as data normalized to control CFA values.

### RNA extraction and quantitative real-time PCR

Striata were dissected in RNAse-free conditions. Total RNA was extracted according to the standard miRNeasy Micro kit protocol (Qiagen). Next, 350 ng of total RNA was reverse-transcribed using High-Capacity cDNA Reverse Transcription Kit (Applied Biosystem), and 10 ng of complementary DNA (cDNA) was amplified with SensiMix SYBR Hi-Rox Kit (Bioline; Meridian Life Science) in triplicate using the Applied Biosystem 7900HT Fast Real-Time PCR system. Messenger RNA (mRNA) relative quantification was performed using the comparative cycle threshold (2^−ΔΔCt^) method. β-actin was used as endogenous control.

Primer sequences:

Ionized calcium binding adaptor protein-1 (Iba-1, NM_019467): 5′-GACAGACTGCCAGCCTAAGACAA-3′ (sense), 5′-CATTCGCTTCAAGGACATAATATCG-3′ (antisense);

β-ACTIN (NM_007393): 5′-CCTAGCACCATGAAGATCAAGATCA-3′ (sense), 5′-AAGCCATGCCAATGTTGTCTCT-3′ (antisense).

For CD3^+^ mRNA detection, 17.5 ng of the same cDNA was amplified with SensiMix II Probe (Bioline; Meridian Life Science) by using TaqMan gene expression assays (ID Mm00599684_g1 and ID Mm00607939; Applied Biosystem).

### Immunofluorescence and confocal microscopy

Mice from two different immunization experiments were deeply anesthetized and intracardially perfused with ice-cold 4 % paraformaldehyde. Brains were post-fixed for 2 h and equilibrated with 30 % sucrose at least one overnight. Immunofluorescence was performed as in Mandolesi et al. [8] and Gentile et al. [22]. The following primary antibodies were used: rabbit anti-Iba1 (1:750; Wako Chemicals, USA), rabbit anti-GFAP (1:500; DAKO), rat anti-CD3 (1:300; AbD Serotec). Secondary antibodies used are as follows: Alexa Fluor-488 (1:200; Life Technologies) and Cy3-conjugated donkey anti-rabbit or anti-rat (1:200; Jackson ImmunoResearch). 4′,6-diamidino-2-phenylindole (DAPI; 0.01 mg/ml) was used to visualize nuclei. All images were acquired using a LSM7 Zeiss confocal laser-scanner microscope (Zeiss, Göttingen, Germany) and processed by NHI ImageJ software [8, 22].

### Immunohistochemistry

A section every third (for a total of eight sections per animal) throughout the rostro-caudal extent of the striatum was selected for staining and examination. Freshly cut 30-μm serial sections, after blocking of nonspecific staining with 10 % normal donkey serum, were incubated overnight with a rabbit polyclonal antibody against parvalbumin (PV) (1:1000; Immunological Science), then with a biotinylated rabbit secondary antibody (1:500; Vector Laboratories, Burlingame, CA, USA) for 1 h, and finally with Extravidin (1:1000; SIGMA) for 1 h. Reaction was developed in a freshly prepared diaminobenzidine (DAB)/H_2_O_2_ (DAB tablet, SIGMA). Sections were mounted on glass slides and coverslipped under Eukitt.

### Stereology

Quantitative observations were limited to the dorsal striatum of the left hemisphere. Using the Stereo Investigator System (MicroBrightField Europe e.K., Magdeburg, Germany) composed of a Zeiss Axioimager.M2 microscope and MicroBrightField’s Stereo Investigator software package, an optical fractionator, stereological design was applied to obtain unbiased estimates of total PV+ cells. Sampling grids and magnifications were adjusted to obtain a relatively constant number of cells sampled and a coefficient of error (CE Gunderson) of ≤0.1. A tri-dimensional optical dissector counting probe (*x*, *y*, *z* dimension of 200 × 200 × 25 μm, respectively) was applied. Counts were performed using a ×20 objective.

Total number was estimated according to the following formula:

$$ \mathrm{N}=\sum \mathrm{Q}\times 1/\mathrm{s}\mathrm{s}\mathrm{f}\times 1/\mathrm{a}\mathrm{s}\mathrm{f}\times 1/\mathrm{t}\mathrm{s}\mathrm{f}. $$

where ΣQ represents the total number of neurons counted in all optically sampled fields of the dorsal striatum, ssf is the section sampling fraction, asf is the area sampling fraction, and tsf is the thickness sampling fraction.

### Cell culture supernatant Luminex assay

The BV2 immortalized murine microglial cell line was pre-treated for 1 h with siponimod 0.1 μM in dimethyl sulfoxide (DMSO), before incubation with tumor necrosis factor (TNF; 200 U/ml; Milteny Biotec); control cells received equal volume of DMSO (*n* = 3 per condition). The 24-h conditioned media was assayed for IL-6 and RANTES by Luminex assay, according to the manufacturer instructions (R&D systems). The plate was read on a Luminex-200 instrument (Luminex Corp., Austin, TX). Concentrations were calculated by using a standard 5P-logistic weighted curve generated for each target and expressed as picograms per milliliter (pg/ml).

### Statistical analysis

For each type of experiment, at least three mice per group were employed. Throughout the text, “*n*” refers to the number of animals, except for electrophysiology, where it means the number of cells. Data were presented as the mean ± S.E.M. The significance level was established at *p* < 0.05. Statistical analysis was performed using unpaired Student’s *T* test for comparisons between two groups and non-parametric Mann-Whitney test, where needed. Multiple comparisons were analyzed by one-way ANOVA for independent measures followed by Tukey’s HSD or Newman-Keuls.

## Results

### Intracerebroventricular injection of 0.45 μg/day siponimod ameliorates clinical score of EAE mice without affecting peripheral CD3^+^ cell count

**Fig. 1:**
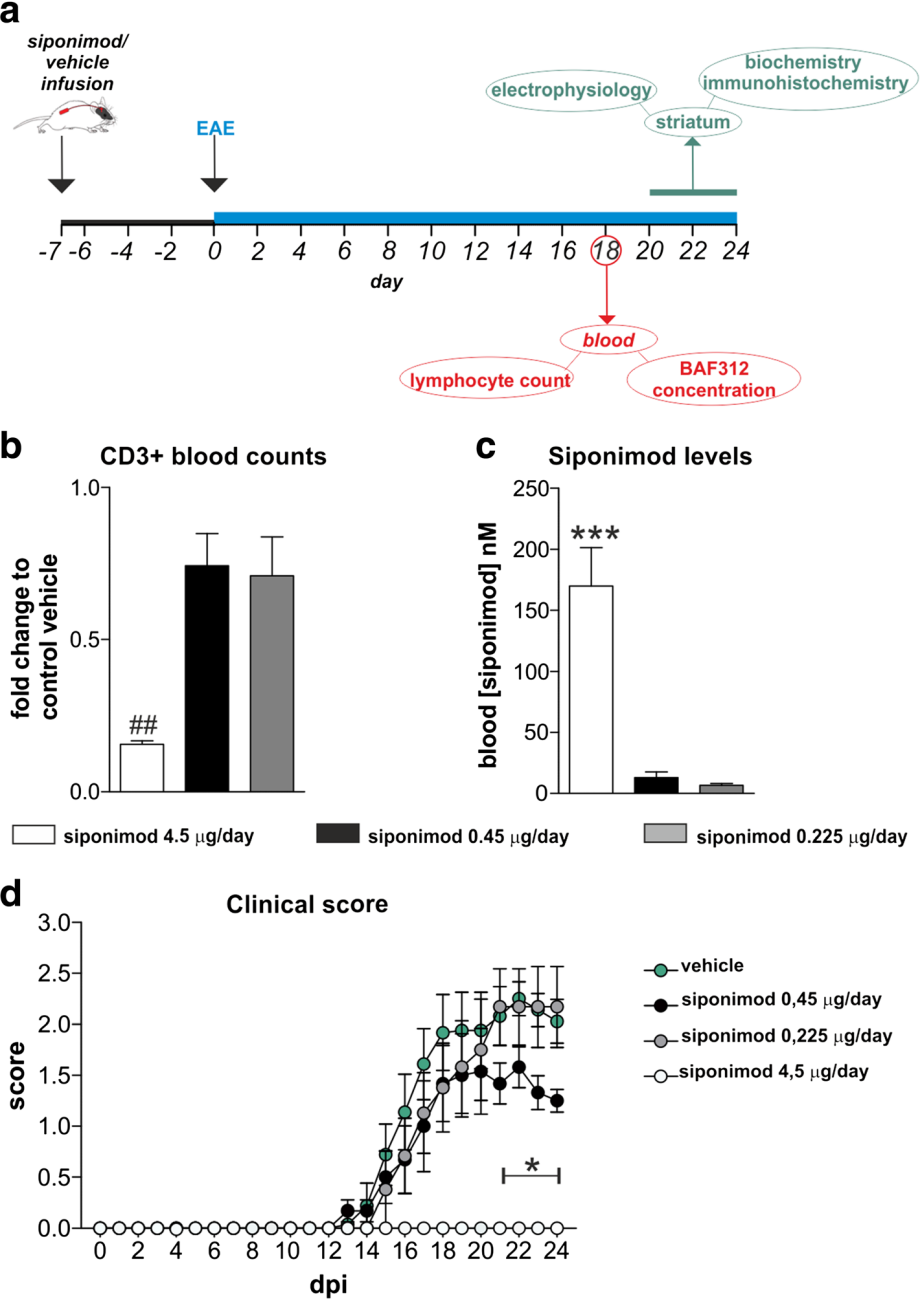
Intracerebroventricular administration of 0.45 μg/day siponimod attenuates EAE motor deficits without inducing peripheral lymphocyte depletion. **a** Schematic representation of the experimental design used in the study. Minipump implantation and siponimod/vehicle release preceded the induction of EAE by 1 week. All examinations were performed on animals during the peak of the symptomatic phase of the disease 18–24 dpi, both in the peripheral blood samples and in the striatum of the animals. **b** CD3^+^ lymphocytes were counted in the peripheral blood of EAE mice receiving vehicle or different siponimod dosages: significant reduction was observed for the 4.5 μg/day dosage. Siponimod 4.5 μg/day vs vehicle ##*p <* 0.01 unpaired *T* test; siponimod 0.45 μg/day vs vehicle *p >* 0.05 unpaired *T* test; siponimod 0.225 μg/day vs vehicle *p >* 0.05 unpaired *T* test. **c** Siponimod concentrations were determined in peripheral blood samples of EAE mice receiving intracerebroventricular release of different siponimod dosages: the highest concentration (4.5 μg/day) used was significantly higher compared to both 0.450 μg/day and 0.225 μg/day, while there were no statistically differences between the other two dosages. Tukey’s post hoc ***p < 0.001. **d** Representative clinical course of EAE mice treated with three different siponimod dosages: EAE disease progression is strongly affected by 4.5 μg/day dosage during the symptomatic phase of the disease and significantly attenuated by 0.45 μg/day concentration in the dpi range of 18–24. Daily statistical significance was evaluated by non-parametric Mann-Whitney test

In order to assess whether siponimod has direct neuroprotective effects, the drug was delivered directly into the brain by means of continuous intracerebroventricular infusion, starting 1 week before the induction of EAE. During the acute phase of the disease, several biochemical and electrophysiological analyses were performed on both the peripheral and the CNS compartment (Fig. 1a, timeline of the experimental design).

First, we tested three dosages of the drug (4.50, 0.450, and 0.225 μg/day) for their possible impact on peripheral T lymphocytes: siponimod is lipophilic and therefore able to cross the blood brain barrier (BBB). At 18 dpi, during the symptomatic phase of the disease, we counted the total number of CD3^+^ lymphocytes in peripheral blood samples of EAE mice receiving different dosages of siponimod or vehicle: while the highest dose of siponimod (4.5 μg/day; *n* = 3) elicited a strong reduction of T lymphocytes in the blood of EAE mice compared to EAE-vehicle mice (*n* = 4, *p* < 0.001; unpaired *T* test), the intermediate (0.450 μg/day; *n* = 6) and the lowest (0.225 μg/day; *n* = 4) dosages of siponimod induced a slight and not significant drop in T lymphocyte counts with respect to EAE-vehicle mice (unpaired *T* test; *p* > 0.05) (Fig. 1b). This result was consistent with siponimod concentrations found in peripheral blood samples (Fig. 1c): intracerebroventricular release of 4.5 μg/day siponimod (*n* = 4) resulted in a high amount of the drug in the peripheral blood (169.9 ± 31.49 nM), significantly different from that measured in the blood of mice receiving 0.45 μg/day (*n* = 3) and 0.225 μg/day (*n* = 5; one-way ANOVA *p* < 0.01; Tukey’s post hoc comparisons: 4.5 μg/day vs 0.45 and 0.225 μg/day, *p* < 0.001). The concentrations of siponimod detected in mice receiving 0.45 or 0.225 μg/day were detectable, but low (13 ± 4.7 nM and 6.6 ± 1.5 nM for the 0.45 and 0.225 μg/day groups, respectively). These levels are not expected to have major effects on peripheral lymphocytes in the majority of animals as confirmed by minor effects observed on peripheral lymphocyte count.

From a clinical point of view, such preventive and CNS-directed treatment reduced motor disability associated to EAE in a dose-dependent manner (Fig. 1d). The highest dose of siponimod used in this study fully inhibited EAE development in treated mice compared to EAE-vehicle (EAE-siponimod *n* = 5, EAE-vehicle *n* = 9; from day 15 to 24 *p* < 0.001, non-parametric Mann-Whitney). This result, together with that shown in Fig. 1b, c, suggests that siponimod effects on peripheral lymphocytes mediate its disease-modifying activity, when administered at this daily concentration. Interestingly, while the 0.450 μg/day (*n* = 6) and the 0.225 μg/day (*n* = 6) doses exerted comparable effects on peripheral lymphocytes, only the former significantly ameliorated the disease severity (*p* < 0.05, non-parametric Mann-Whitney) in the dpi range of 21–24 of the disease compared to EAE-vehicle (*n* = 9) (Fig. 1d).

Bringing together these data, we decided to carry out the study using the 0.450 μg/day dose to better dissect the neuroprotective action of siponimod.

### Siponimod icv treatment reduces gray matter inflammation in EAE mice

Activation of microglia and astrocytes are hallmarks of both MS and EAE [23]. Both cell populations have been demonstrated to sustain inflammation within the CNS during autoimmune attack through antigen presentation and/or cytokine/chemokine secretion [24–26]. Moreover, we previously demonstrated that microglia and astroglia together with infiltrating lymphocytes play a pivotal role in inducing the synaptic alterations observed in EAE brain [8–10, 15, 27].

**Fig. 2:**
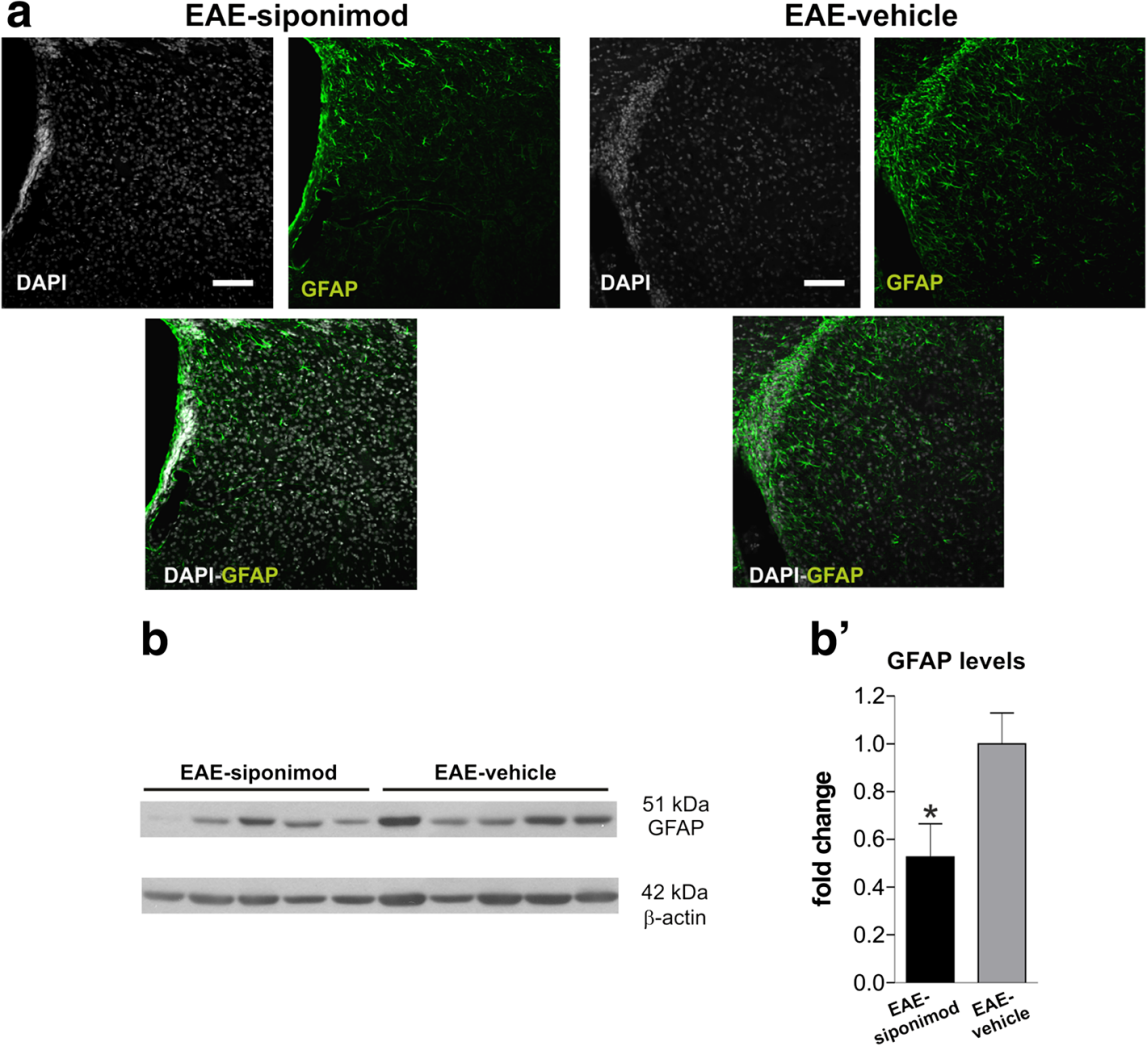
CNS-directed delivery of siponimod reduces astrogliosis in the EAE striatum. **a** Confocal microscopy images depict the striatum of EAE-vehicle and siponimod mice stained for the astrocyte marker GFAP (green fluorescence, counterstained with DAPI, *gray*). Expression of GFAP is markedly reduced in EAE-siponimod striatum compared to EAE-vehicle striatum (scale bar: 100 μm). The images are representative of stained sections from three animals per groups from two immunizations. The panel in **b** shows western blot comparing EAE-siponimod and EAE-vehicle striatal extracts probed for anti-GFAP antibody. Densitometric analysis of the bands in **b’** reveals reduced GFAP content, relative to β-actin, in EAE-siponimod lysates compared to EAE-vehicle. WB data are normalized to EAE-vehicle values. Unpaired *T* test was used for two-group analysis. Values are means ± SEM, *p < 0.05

We investigated by immunohistochemistry the effect of siponimod on brain inflammatory reaction during EAE. Coronal striatal slices were probed for the GFAP, a marker upregulated during astrocyte activation. As depicted in Fig. 2a, a substantial reduction of astrogliosis was observed in EAE-siponimod striatum (green fluorescence for GFAP staining, DAPI counterstaining in gray) compared to EAE-vehicle. Qualitative data of confocal microscopy images were confirmed by western blot analysis of protein lysates from EAE-siponimod striatum compared to EAE-vehicle striatum: GFAP levels, normalized to β-actin, were reduced by 50 % in the striatum of EAE mice treated with siponimod (Fig. 2b, b’; *n* = 5 for EAE-vehicle, *n* = 5 for EAE-siponimod; *p* < 0.05, unpaired *T* test).

**Fig. 3:**
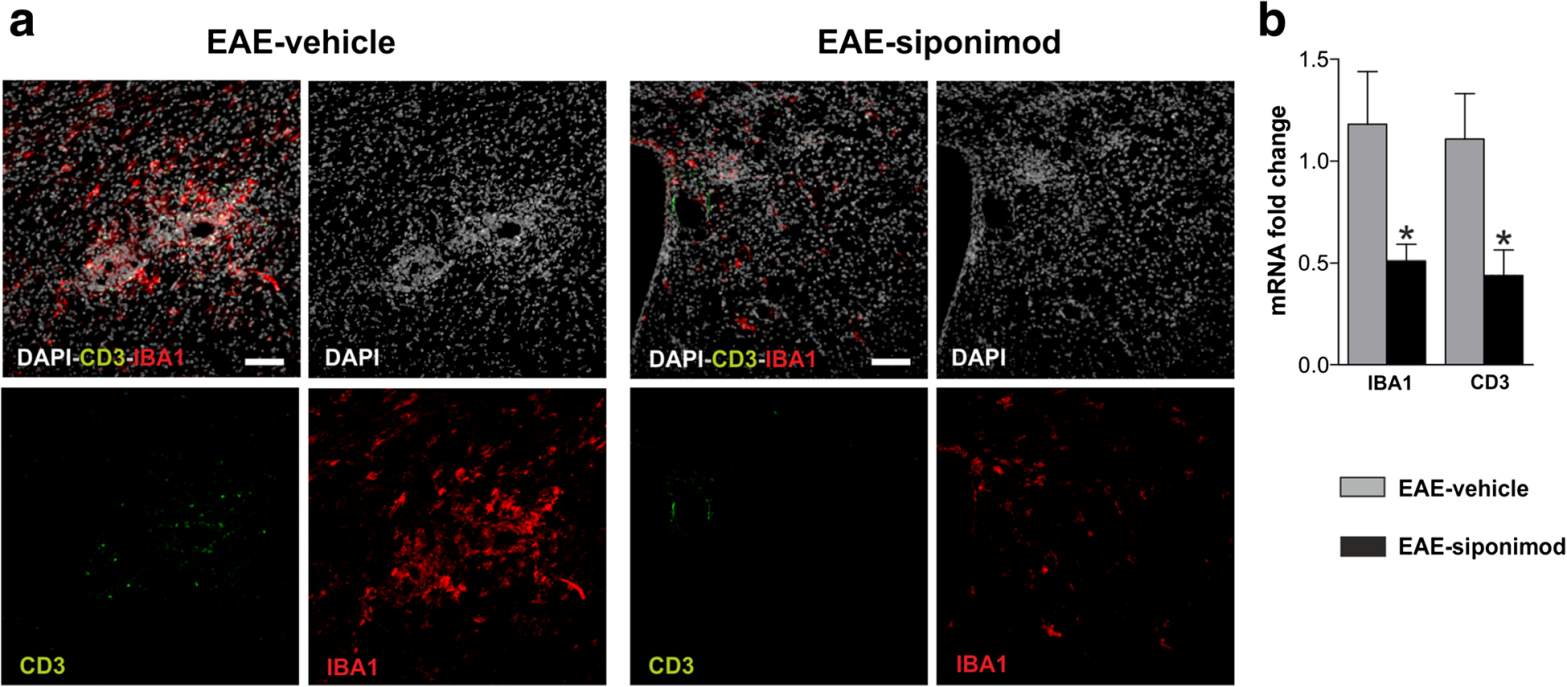
Attenuated microgliosis and reduced lymphocyte infiltration characterizes the striatum of EAE mice treated with siponimod. **a** Double immunostaining of coronal striatal sections (in *gray* DAPI nuclei) showing expression of Iba1-positive microglia/macrophage cells (*red*) and of CD3 (*green*) in EAE-siponimod and EAE-vehicle mice. Only few lymphocytes could be detected in the striatum of mice treated with siponimod, and microgliosis is visibly reduced in the same animals. Scale bar: 100 μm. **b** The levels of IBA1 and CD3 mRNA were quantified by qRT-PCR in the striatum of EAE-siponimod versus EAE-vehicle mice using β-actin as internal control. The histogram shows that both IBA1 and CD3 mRNA were significantly reduced in the striatum of siponimod-treated mice. Statistical significance was evaluated by unpaired *T* test: *p < 0.05

Microgliosis was assessed by immunofluorescence and qPCR. As shown in Fig. 3a, the staining for the ionized calcium binding adaptor protein-1 (IBA1), marker of microglia/macrophage, is more pronounced in EAE-vehicle than in EAE-siponimod striatum (red fluorescence, counterstaining with DAPI in gray). Moreover, in the same slices, icv treatment with siponimod reduced the number of infiltrating lymphocytes compared to the icv-vehicle control group (Fig. 3a, green fluorescence). Quantitative analysis of IBA1 and CD3 transcripts by qPCR confirms reduced microglia activation and lymphocyte infiltration in the striatum of EAE mice receiving siponimod in comparison to EAE-vehicle (Fig. 3b; *n* = 4 for EAE-vehicle, *n* = 5 for EAE-siponimod; *p <* 0.05, unpaired *T* test).

### In vitro application of siponimod reduces IL-6 and RANTES release by TNF-treated microglial cells

Microglia have been clearly involved in neuronal damage and T lymphocyte recruitment in the brain, by releasing pro-inflammatory cytokines and chemokines, like IL-6 and RANTES, which have been implicated in EAE and MS pathogenesis [28–32].

**Fig. 4:**
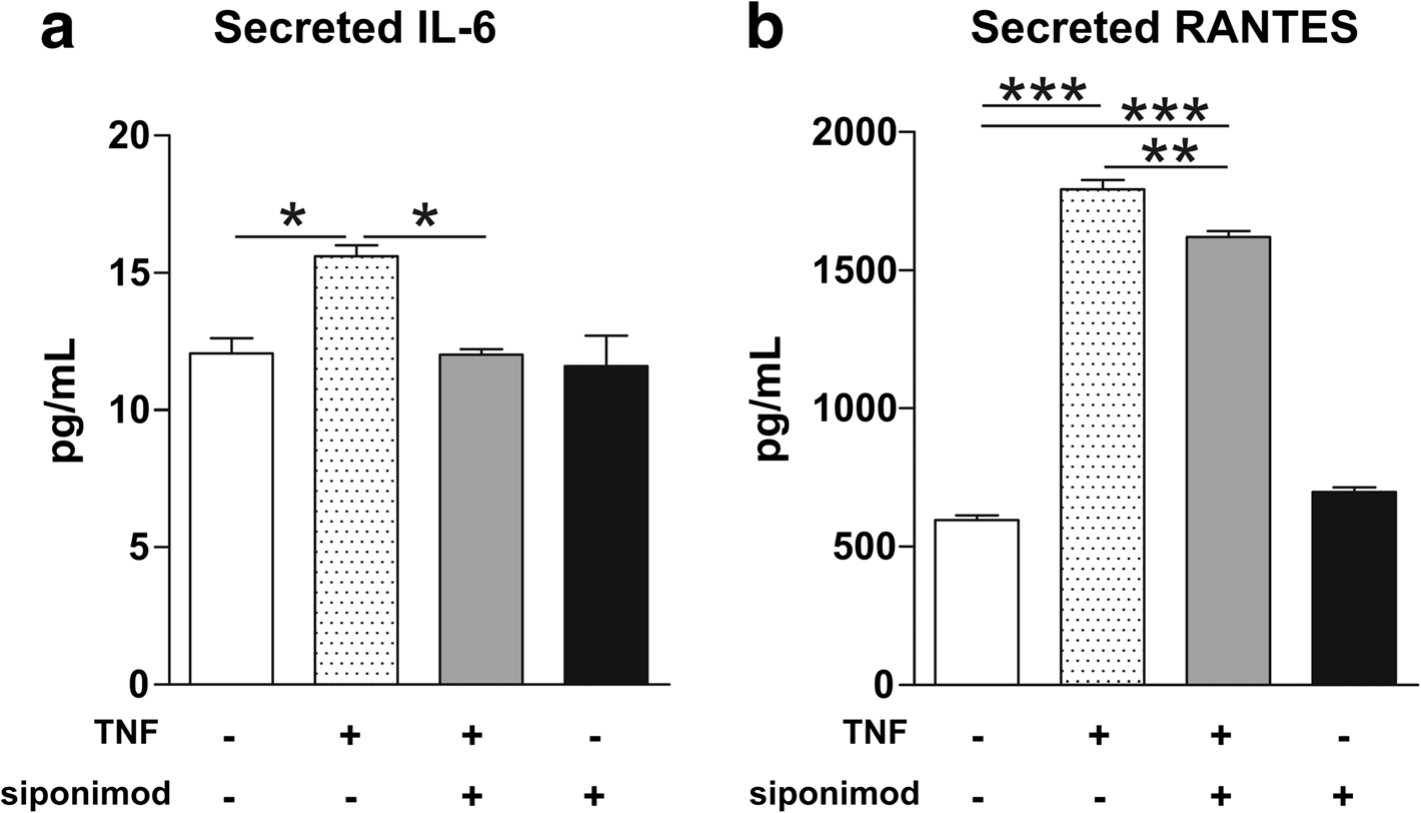
In vitro siponimod application to activated BV2 microglial cell reduces IL-6 and RANTES release. BV2 cells were pre-treated with siponimod prior to stimulation with TNF. The levels of secreted IL-6 (**a**) and RANTES (**b**) were measured by Luminex assay on collected media. Application of siponimod to non-activated cells does not modulate the secretion of both molecules. Data were expressed as picograms per milliliter (pg/ml) and were analyzed by one-way ANOVA, followed by Tukey’s post hoc test. *p < 0.05, **p < 0.01, ***p < 0.001

We tested the hypotheses that siponimod might reduce microglia activation. To this aim, we used an in vitro model of microglia activation. BV2 microglial cells were pre-treated with 0.1 μM siponimod prior to 24-h stimulation with TNF. By Luminex assay (Fig. 4a), we found that siponimod prevented the release of IL-6 induced by TNF stimulation of BV2 cells (one-way ANOVA: control cells 12.06 ± 0.55 pg/ml vs TNF 15.61 ± 0.40 pg/ml, *p <* 0.05 Tukey’s post hoc; TNF + siponimod 12.01 ± 0.21 pg/ml vs TNF, *p <* 0.05 Tukey’s post hoc). Siponimod in the absence of TNF did not alter the basal secretion of IL-6 (control cells vs vehicle + siponimod 11.6 ± 1.11 pg/ml, *p >* 0.05 Tukey’s post hoc).

TNF induced a strong release of RANTES in cell culture supernatant of BV2 cells (one-way ANOVA: control cells 596.2 ± 17.4 pg/ml vs TNF 1794 ± 33.65 pg/ml, *p <* 0.001 Tukey’s post hoc; Fig. 4b). Siponimod reduced the amount of RANTES released by TNF-stimulated cells (TNF vs siponimod + TNF 1621 ± 21.67 pg/ml, *p <* 0.01 Tukey’s post hoc; siponimod + TNF vs control *p <* 0.01 Tukey’s post hoc) (Fig. 4b). As for IL-6, siponimod did not alter the release of RANTES by BV2 microglial cells in the absence of TNF (control vs siponimod-TNF 698.4 ± 16.44 pg/ml, *p >* 0.05, Tukey’s post hoc).

### Siponimod treatment recovers GABAergic transmission alterations in EAE

We next asked whether siponimod might improve the synaptic transmission alterations typical of EAE brains [11], by recording both the glutamatergic and the GABAergic currents from striatal MSNs.

**Fig. 5:**
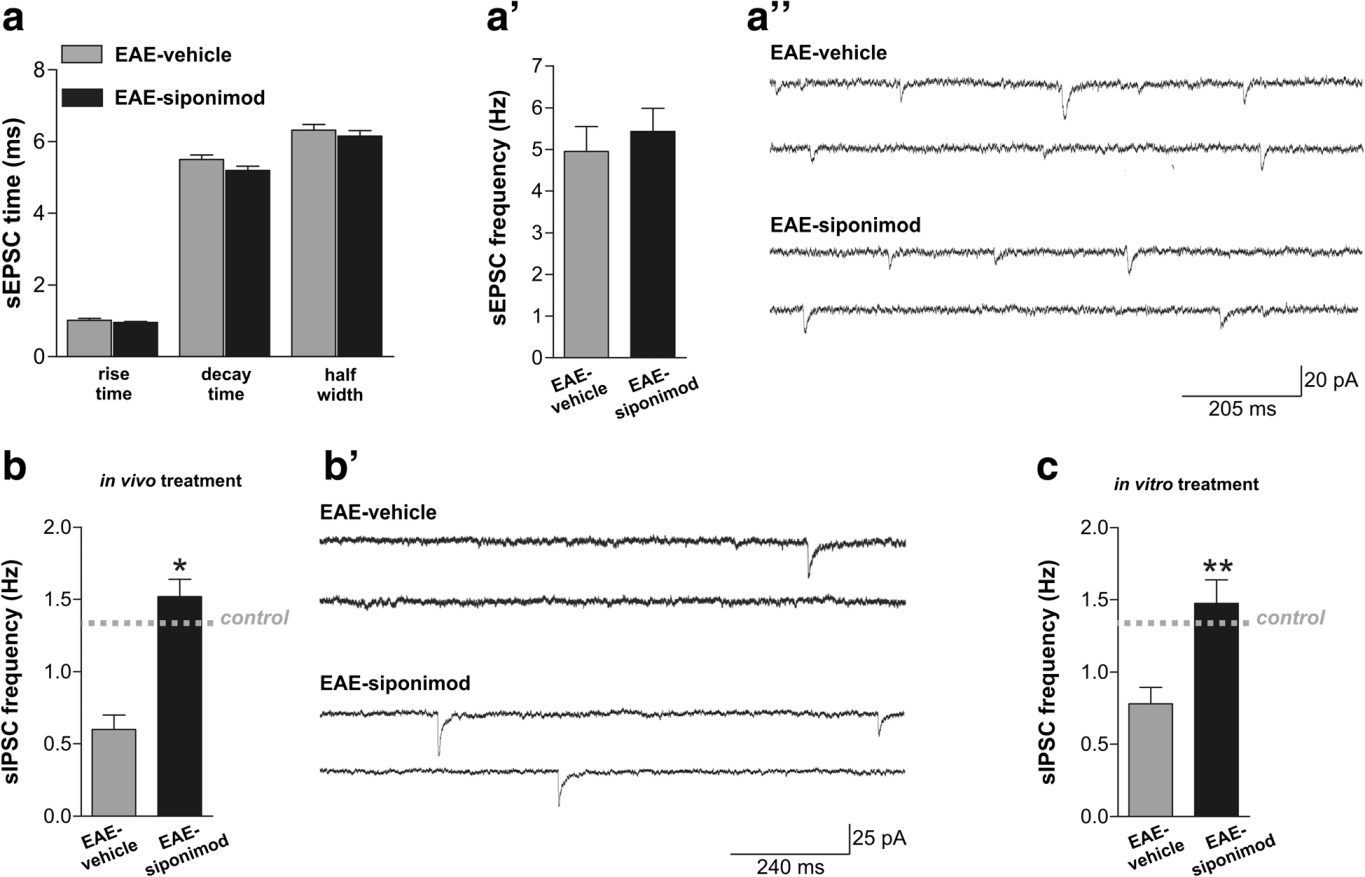
Siponimod treatment corrects GABAergic defects in EAE striatum. **a** The glutamatergic transmission was recorded from MSNs of EAE-vehicle and EAE-siponimod mice. The analysis of the increased kinetic properties (rise time, decay time, and half width) of sEPSCs showed that there were no differences between the two groups (unpaired *T* test, *p >* 0.05). **a’** The frequencies of the glutamatergic currents, increased in EAE striatum, were not corrected by siponimod (unpaired *T* test, *p > 0.05). **a”** Electrophysiological traces representative of glutamatergic currents recorded from MSNs of EAE-vehicle and EAE-siponimod mice. **b** Siponimod restores normal GABAergic frequencies in the striatum of acute phase EAE mice (unpaired *T* test,*p < 0.05). **b’** Electrophysiological traces representative of GABAergic currents recorded from MSNs of EAE-vehicle and EAE-siponimod mice. **c** The frequencies of the GABAergic currents, reduced in EAE striatum, were completely rescued after 1 h of siponimod incubation in EAE slices (unpaired *T* test, **p < 0.01)

We previously demonstrated that both the presynaptic (frequency) and post-synaptic (kinetic) properties of the spontaneous glutamatergic currents (sEPSCs) are altered in the striatum of EAE mice during the acute phase of the disease [15]. Here, we first investigated the effect on sEPSCs after in vivo treatment with siponimod 0.45 μg/day on sEPSCs, by recording striatal slices in the dpi range of 20–24. Siponimod did not correct neither the kinetic parameters of rise time, decay time, and half width (Fig. 5a–a”) nor the frequencies (Fig. 5a’–a”) of EAE sEPSC (*n* = 8 for both groups; *p >* 0.05, unpaired *T* test for all comparisons).

Conversely, siponimod icv treatment significantly ameliorated GABAergic defects in the striatum of EAE mice, by increasing GABA frequencies, which are considerably reduced in the symptomatic phase of the disease [6]. Siponimod restored normal values of frequency of spontaneous inhibitory post-synaptic currents (sIPSCs) (Fig. 5b, b’; EAE-vehicle 0.61 ± 0.11 Hz, *n* = 10; EAE-siponimod: 1.52 ± 0.12 Hz, *n* = 8; *p <* 0.05, unpaired *T* test), suggesting that siponimod might have neuroprotective effects by preventing GABAergic transmission deficits.

Furthermore, in order to better explain the protective role exerted by siponimod on GABA synapses, we incubated EAE slices taken during the symptomatic phase of the disease (20–25 dpi) with siponimod (1 μM) or vehicle (DMSO) for 1 h. The in vitro treatment with siponimod was able to rescue the GABAergic impairment induced by EAE (Fig. 5c). The sIPSC frequency was higher in EAE-siponimod slices (1.47 ± 0.61 Hz, *n* = 8) in comparison to EAE untreated slices (0.78 ± 0.11 Hz; *n* = 8; *p <* 0.01, unpaired *T* test), reaching values indistinguishable from control slices. These data support the idea that siponimod exerts its central protective role likely by reducing resident immune cell activation.

### Siponimod promotes PV+ interneuron survival

GABAergic interneurons, together with axon collaterals from MSNs themselves, are the main source of GABA inputs to striatal MSNs [33, 34]. The loss of parvalbumin-positive (PV+) GABAergic interneurons together with the noxious effects of pro-inflammatory cytokines has been proposed as major determinant of the reduced GABAergic tone in the striatum of EAE mice [6]. Therefore, we assessed whether siponimod could prevent PV+ interneuron death in the striatum of EAE mice.

**Fig. 6:**
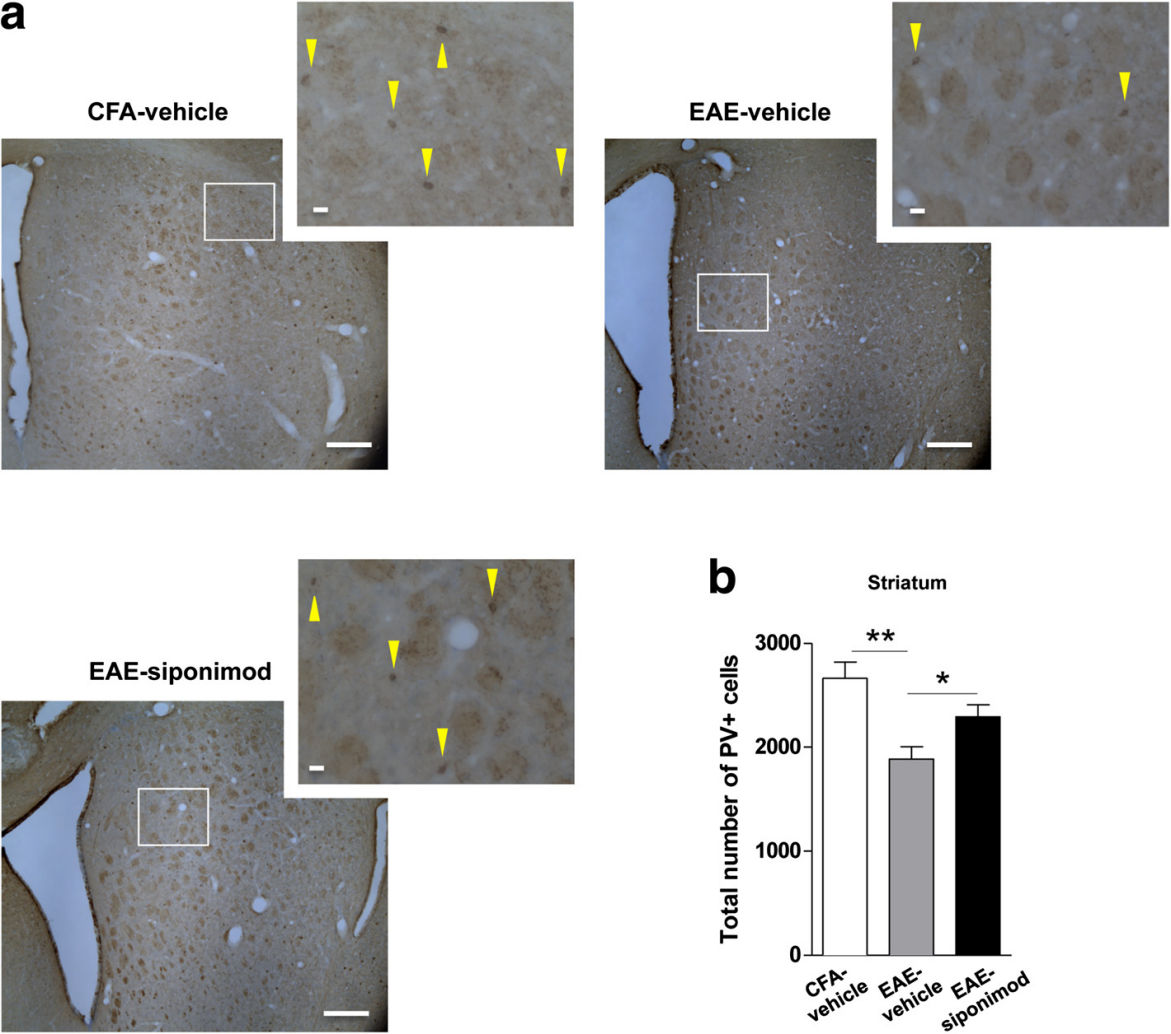
Brain infusion of siponimod promotes the survival of PV+ GABAergic interneurons during the course of EAE. **a** Low magnification images (×5 objective) of coronal striatal sections from control CFA-vehicle, EAE-vehicle, and EAE-siponimod showing area selected for PV counting on serial sections (dorsal striatum) and DAB-immunolabeled interneurons (scale bar: 200 μm): at high magnification, the density of PV+ cells is visibly reduced in EAE-vehicle compared to that in CFA-vehicle with a partial recovery in EAE-siponimod slices. *Yellow arrows* indicate PV-labeled cells (scale bar: 20 μm). **b** Histogram represents the quantitative stereological counts of PV+ cells performed on striatal slices: the intracerebroventricular treatment with siponimod significantly increases the total number of PV+ cells in the dorsal left striatum of EAE mice. Data are expressed as mean ± S.E.M. Statistical comparisons showed in the graph were calculated with Newman-Keuls post hoc: CFA-vehicle vs EAE-vehicle **p < 0.01; CFA-vehicle vs EAE-siponimod *p >* 0.05; EAE-vehicle vs EAE-siponimod*p < 0.05

To avoid possible interference of the damage caused by the cannula inserted in the right lateral ventricle, we performed unbiased stereological counts in the left dorsal striatum of EAE mice. In Fig. 6a, there are representative images of coronal slices stained with anti-parvalbumin antibody. This experimental approach confirmed the reduction of the total number of PV+ neurons in EAE-vehicle (1885 ± 117.7, *n* = 5) compared to control CFA-vehicle striatum (2664 ± 156.6, *n* = 3) and, more interestingly, showed a recovery induced by siponimod (2293 ± 115.3, *n =* 5; one-way ANOVA *p <* 0.01; Newman-Keuls post hoc comparisons: CFA-vehicle vs EAE-vehicle, *p <* 0.01; CFA-vehicle vs EAE-siponimod, *p >* 0.05; EAE-vehicle vs EAE-siponimod, *p <* 0.05; Fig. 6b).

These data indicate that siponimod prevents GABAergic interneuron loss in the striatum of symptomatic EAE mice.

## Discussion

Discovering drugs effective in progressive forms of MS is a hard challenge for both researchers and physicians working in the field of MS. Promoting neuroprotection is a promising therapeutic strategy in the light of the relevant contribution of neurodegeneration to MS pathogenesis and progression [2]. GM damage, including neuron and synaptic loss, is currently considered as the major contributor to both cognitive impairment and motor disability in MS patients [35, 36]. Recently, it has been highlighted that inflammation-driven synaptic abnormalities are pathological hallmarks of both EAE and MS brains: the sustained imbalance between glutamatergic and GABAergic transmission, occurring independently of demyelination and axonal loss, is supposed to induce excitotoxic neurodegeneration [11]. Preventing such inflammatory neurodegenerative mechanisms is a novel and poorly explored therapeutic strategy.

In the present study, we investigated whether siponimod could be neuroprotective in EAE mice, by limiting striatal synaptopathy. Major findings of this study are that the GABAergic transmission alterations typical of the EAE striatum are rescued by siponimod treatment, likely due to reduced local inflammatory reaction and to increased survival of PV+ interneurons, which are the main GABAergic input to MSNs. In an attempt to discriminate the peripheral from the central effects of siponimod, we used a direct and preventive CNS administration of the drug, choosing the concentration of siponimod able to ameliorate the disease severity, but with minimal impact on blood lymphocyte count.

Adjusting the administration dose was our primary goal to avoid that the observed beneficial effects could be secondary to its peripheral immunomodulatory action. In fact, by virtue of its lipophilic nature, siponimod easily crosses the BBB reaching peripheral tissues. Not surprisingly, the highest dosage (4.5 μg/day) tested in this study resulted in significant blood exposure of siponimod and a strong reduction of T cells in the blood, likely as a result of retention in lymph nodes, and completely prevented EAE development. The lower dosages (0.45 and 0.225 μg/day) had both a minimal impact on peripheral lymphocyte count with siponimod blood levels reaching only low nanomolar range. Since the 0.225 μg/day dose had no effect on the clinical score, we chose the 0.45 μg/day dosage due to its beneficial effect on disease severity, even if this was limited to the peak of the acute phase (20–24 dpi). However, since the clinical symptoms are mainly due to lesions in the spinal cord and since we expect a gradient of the compound between the site of administration and the site of the spinal cord lesions, a lack of strong clinical efficacy does not rule out more potent effects at the proximity of the administration site. Moreover, although we cannot exclude any peripheral effect of siponimod, we might assume that the observed recovery in clinical score could be linked to central effects of this drug. Accordingly, this treatment induced attenuation of microgliosis and astrogliosis with reduced number of infiltrating CD3^+^ lymphocytes in the striatum of EAE mice during the symptomatic phase of the disease. A possible explanation for the low number of infiltrating lymphocytes could be a reduced inflammatory reaction in the brain, caused by the preventive and central treatment with siponimod, likely promoting a different tissue distribution of T cells compared to EAE untreated mice.

Indeed, CNS-resident cells, such as microglia, astroglia, oligodendrocytes, and neurons, express sphingosine receptors, although with some differences in the receptor subunit composition [21]. Most of the anti-inflammatory effects of S1PR agonists, like fingolimod and siponimod, in the CNS have been associated to their action on astroglia and microglia [37, 38]. Microglia activation occurs early in the EAE brain and spinal cord, before the appearance of motor deficits [25, 39, 40]. By secreting a number of pro-inflammatory cytokines and chemotactic factors, microglia are involved in the amplification of the inflammatory reaction and the recruitment of monocyte-blood-born cells and T cells into the brain. In our experimental paradigm of preventive and central treatment with siponimod, siponimod may prevent the chain of noxious events triggered by microglia in the EAE brain, reducing MOG-specific lymphocyte recruitment. To prove this hypothesis, we used an in vitro simplification of the in vivo experimental approach: we pre-treated microglial cells with siponimod prior to activation with TNF. We found that siponimod reduced microglial release of IL-6 and RANTES, some of the most relevant cytokines/chemokines involved in EAE/MS pathogenesis [28–32]. Indeed, although IL-6 levels in the CSF of MS patients have reported to be unaltered, likely indicating that IL-6 is not a specific signature of the disease [41], it can be hypothesized that this cytokine has a local role in the modulation of the inflammatory reaction, since its expression was detected in glial cells of MS lesions [42]. Conversely, it is worth noting that RANTES levels have been found increased in the CSF of MS patients [43] and, most notably, they correlated with inflammation and synaptic excitability [44].

We have recently demonstrated that inflammation causes an imbalance between the glutamatergic and the GABAergic transmission in the EAE striatum [6, 15]. Notably, such alterations are putative therapeutic targets, and some drugs currently used in MS treatment have already been found able to interfere with the chain of events leading to such dysregulation [45–47]. Of note, fingolimod, given systemically and starting the day of immunization, was shown to strongly ameliorate clinical deficits and to correct the glutamatergic transmission alterations of EAE striatum [45]. Here, we demonstrated that siponimod rescued GABAergic transmission defects, without improving the glutamatergic transmission in MSNs. Such differences between the results obtained with the two S1P modulators probably stems from the way of administration of the drug and the dose/drug exposure. The oral delivery of fingolimod likely prevented T lymphocytes from migrating into the brain, thus explaining the marked effect on disease progression and the excitatory currents. Notably, even if in a small number, infiltrating T cells, which have been highly involved in glutamatergic alteration in the striatum of EAE mice [15] may be able to affect excitatory transmission.

Furthermore, an acute exposure (1 h) of siponimod to slices of EAE mice reproduced the same effect of the in vivo treatment on GABA transmission, reinforcing the idea that the compound may improve the inhibitory tone in the striatum of EAE mice by interacting with resident immune cells.

In the present study, we observed that icv treatment with siponimod recovered the frequencies of the sIPSCs in EAE striatum. This specific impairment of the GABAergic neurotransmission has been linked to inflammation, although a selective cytokine or chemokine has not been recognized, and to the reduced number of GABAergic interneurons contacting MSNs in the EAE striatum [6]. The death of PV+ interneurons is a pathological hallmark of both MS and EAE brains [6, 48], and the mechanisms leading to such degeneration are still unknown. Inflammation is thought to play a role in this neuronal death, which has been observed in other brain inflammatory conditions [49] and in rodent chronic stress paradigms [50].

In the striatum of EAE mice, GABAergic defects occur throughout the disease course [6]. In our study, we found that siponimod promotes the survival of these neurons, thus explaining the recovery of GABAergic neurotransmission, in spite of the potentiated glutamatergic transmission. Further studies are needed to dissect the mechanisms of neuroprotection exerted by siponimod on PV+ interneurons: other mechanisms involving neuroprotective factors, like the brain-derived neurotrophic factor (BDNF), may be implied [51].

It is worth noting that reduced GABA concentrations in the hippocampus and sensory-motor cortex have recently been correlated with physical disability in progressive MS patients [52]. This finding strongly corroborates the role of the inflammatory synaptopathy in MS progression. Interestingly, in our study, the attenuation of the motor symptoms of EAE mice treated with siponimod was observed in the symptomatic phase of the disease in connection to the improved tone of the GABAergic transmission in the striatum, a subcortical brain area involved in motor control [53] and compromised in MS [54].

## Conclusions

In conclusion, the present investigation highlights a neuroprotective activity of siponimod with respect to a relevant feature of neurodegeneration affecting MS brains, the reduced tone of GABAergic transmission.

## Abbreviations

BBB: Blood brain barrier
BDNF: Brain-derived neurotrophic factor
CFA: Complete Freund’s adjuvant
CNS: Central nervous system
DAB: Diaminobenzidine
DAPI: 4′,6-Diamidino-2-phenylindole
DMSO: Dimethyl sulfoxide
dpi: Day post-immunization
EAE: Experimental autoimmune encephalomyelitis
GABA: Gamma-amino butyric acid
GFAP: Glial fibrillary amino acid protein
GM: Gray matter
IBA1: Ionized calcium binding adaptor protein-1
icv: Intracerebroventricular
IL: Interleukin
MOG: Myelin oligodendrocyte glycoprotein
MRM: Multiple reaction monitoring
MS: Multiple sclerosis
MSN: Medium spiny neuron
PBS: Phosphate buffer solution
PPMS: Primary progressive MS
PV: Parvalbumin
qPCR: Quantitative-polymerase chain reaction
RANTES: Regulated on activation, normal T cell expressed and secreted
RRMS: Relapsing-remitting MS
S1P: Sphingosine-1-phosphate receptor
sEPSC: Spontaneous excitatory post-synaptic currents
sIPSCs: Spontaneous inhibitory post-synaptic currents
SPMS: Secondary progressive MS
TBS: Tris buffered solution
TNF: Tumor necrosis factor
WB: Western blot
